# Open Babel: An open chemical toolbox

**DOI:** 10.1186/1758-2946-3-33

**Published:** 2011-10-07

**Authors:** Noel M O'Boyle, Michael Banck, Craig A James, Chris Morley, Tim Vandermeersch, Geoffrey R Hutchison

**Affiliations:** 1 Analytical and Biological Chemistry Research Facility, Cavanagh Pharmacy Building, University College Cork, Co. Cork, Ireland; 2 Department of Chemistry, Technische Universität München, Garching D-85747, Germany; 3 eMolecules, Inc., 420 Stevens Ave #120, Solana Beach, CA 92075, USA; 4 Open Babel development team; 5 University of Pittsburgh, Department of Chemistry, 219 Parkman Avenue, Pittsburgh, PA 15217, USA

## Abstract

**Background:**

A frequent problem in computational modeling is the interconversion of chemical structures between different formats. While standard interchange formats exist (for example, Chemical Markup Language) and *de facto *standards have arisen (for example, SMILES format), the need to interconvert formats is a continuing problem due to the multitude of different application areas for chemistry data, differences in the data stored by different formats (0D versus 3D, for example), and competition between software along with a lack of vendor-neutral formats.

**Results:**

We discuss, for the first time, Open Babel, an open-source chemical toolbox that speaks the many languages of chemical data. Open Babel version 2.3 interconverts over 110 formats. The need to represent such a wide variety of chemical and molecular data requires a library that implements a wide range of cheminformatics algorithms, from partial charge assignment and aromaticity detection, to bond order perception and canonicalization. We detail the implementation of Open Babel, describe key advances in the 2.3 release, and outline a variety of uses both in terms of software products and scientific research, including applications far beyond simple format interconversion.

**Conclusions:**

Open Babel presents a solution to the proliferation of multiple chemical file formats. In addition, it provides a variety of useful utilities from conformer searching and 2D depiction, to filtering, batch conversion, and substructure and similarity searching. For developers, it can be used as a programming library to handle chemical data in areas such as organic chemistry, drug design, materials science, and computational chemistry. It is freely available under an open-source license from http://openbabel.org.

## Introduction

The history of chemical informatics has included a huge variety of textual and computer representations of molecular data. Such representations focus on specific atomic or molecular information and may not attempt to store all possible chemical data. For example, line notations like Daylight SMILES [1] do not offer coordinate information, while crystallographic or quantum mechanical formats frequently do not store chemical bonding data. Hydrogen atoms are frequently omitted from x-ray crystallography due to the difficulty in establishing coordinates, and are often ignored by some file formats as the "implicit valence" of heavy atoms that indicates their presence. Other types of representations require specification of atom types on the basis of a specific valence bond model, inclusion of computed partial charges, indication of biomolecular residues, or multiple conformations.

While attempts have been made to provide a standard format for storing chemical data, including most notably the development of Chemical Markup Language (CML) [2-6], an XML dialect, such formats have not yet achieved widespread use. Consequently, a frequent problem in computational modeling is the interconversion of molecular structures between different formats, a process that involves extraction and interpretation of their chemical data and semantics.

We outline for the first time, the development and use of the Open Babel project, a full-featured open chemical toolbox, designed to "speak" the many different representations of chemical data. It allows anyone to search, convert, analyze, or store data from molecular modeling, chemistry, solid-state materials, biochemistry, or related areas. It provides both ready-to-use programs as well as a complete, extensible programmer's toolkit for developing cheminformatics software. It can handle reading, writing, and interconverting over 110 chemical file formats, supports filtering and searching molecule files using Daylight SMARTS pattern matching [7] and other methods, and provides extensible fingerprinting and molecular mechanics frameworks. We will discuss the frameworks for file format interconversion, fingerprinting, fast molecular searching, bond perception and atom typing, canonical numbering of molecular structures and fragments, molecular mechanics force fields, and the extensible interfaces provided by the software library to enable further chemistry software development.

Open Babel has its origin in a version of OELib released as open-source software by OpenEye Scientific under the GPL (GNU Public License). In 2001, OpenEye decided to rewrite OELib in-house as the proprietary OEChem library, so the existing code from OELib was spun out into the new Open Babel project. Since 2001, Open Babel has been developed and substantially extended as an international collaborative project using an open-source development model [8]. It has over 160,000 downloads, over 400 citations [9], is used by over 40 software projects [10], and is freely available from the Open Babel website [11].

## Features

### File Format Support

With the release of Open Babel 2.3, Open Babel supports 111 chemical file formats in total. It can read 82 formats and write 85 formats. These encompass common formats used in cheminformatics (SMILES, InChI, MOL, MOL2), input and output files from a variety of computational chemistry packages (GAMESS, Gaussian, MOPAC), crystallographic file formats (CIF, ShelX), reaction formats (MDL RXN), file formats used by molecular dynamics and docking packages (AutoDock, Amber), formats used by 2D drawing packages (ChemDraw), 3D viewers (Chem3D, Molden) and chemical kinetics and thermodynamics (ChemKin, Thermo). Formats are implemented as "plugins" in Open Babel, which makes it easy for users to contribute new file formats (see Extensible Interface below). Depending on the format, other data is extracted by Open Babel in addition to the molecular structure; for example, vibrational frequencies are extracted from computational chemistry log files, unit cell information is extracted from CIF files, and property fields are read from SDF files.

A number of "utility" file formats are also defined; these are not strictly speaking a way of storing the molecular structure, but rather present certain functionality through the same interface as the regular file formats. For example, the *report format *is a write-only utility format [12] that presents a summary of the molecular structure of a molecule; the *fingerprint format *[13] and *fastsearch format *[14] are used for similarity and substructure searching (see below); the *MolPrint2D *and *Multilevel Neighborhoods of Atoms *formats calculate circular fingerprints defined by Bender *et al. *[15,16] and Filimonov *et al. *[17,18] respectively.

Each format can have multiple options to control either reading or writing a particular format. For example, the InChI format has 12 options including an option "K" to generate an InChIKey, "T <param>" to truncate the InChI depending on a supplied parameter and "w" to ignore certain InChI warnings. The available options are listed in the documentation, are shown in the Graphical User Interface (GUI) as checkboxes or textboxes, and can be listed at the command-line. In fact, all three are generated from the same source; a documentation string in the C++ code.

### Fingerprints and Fast Searching

Databases are widely used to store chemical information especially in the pharmaceutical industry. A key requirement of such a database is the ability to index chemical structures so that they can be quickly retrieved given a query substructure. Open Babel provides this functionality using a path-based fingerprint. This fingerprint, referred to as *FP2 *in Open Babel, identifies all linear and ring substructures in the molecule of lengths 1 to 7 (excluding the 1-atom substructures C and N) and maps them onto a bit-string of length 1024 using a hash function. If a query molecule is a substructure of a target molecule, then all of the bits set in the query molecule will also be set in the target molecule. The fingerprints for two molecules can also be used to calculate structural similarity using the Tanimoto coefficient, the number of bits in common divided by the union of the bits set.

Clearly, repeated searching of the same set of molecules will involve repeated use of the same set of fingerprints. To avoid the need to recalculate the fingerprints for a particular multi-molecule file (such as an SDF file), Open Babel provides a *fastindex *format that solely stores a fingerprint along with an index into the original file. This index leads to a rapid increase in the speed of searching for matches to a query - datasets with several million molecules are easily searched interactively. In this way, a multi-molecule file may be used as a lightweight alternative to a chemical database system.

### Bond Perception and Atom Typing

As mentioned above, many chemical file formats offer representations of molecular data solely as lists of atoms. For example, most quantum chemical software packages and most crystallographic file formats do not offer definitions of bonding. A similar situation occurs in the case of the Protein Data Bank (PDB) format; while standardized [19] files contain connectivity information, non-standard files exist that often do not provide full connectivity information. Consequently, Open Babel features methods to determine bond connectivity, bond order perception, aromaticity determination, and atom typing.

Bond connectivity is determined by the frequently used algorithm of detecting atoms closer than the sum of their covalent radii, with a slight tolerance (0.45 Å) to allow for longer than typical bonds. To handle disorder in crystallographic data (e.g., PDB or CIF files), atoms closer than 0.63 Å are not bonded. A further filtering pass is made to ensure standard bond valency is maintained; each element has a maximum number of bonds, if this is exceeded then the longest bonds to an atom are successively removed until the valence rule is fulfilled.

After bond connectivity is determined, if needed or requested by the user, bond order perception is performed on the basis of bond angles and geometries. The method is similar to that proposed by Roger Sayle [20] and uses the average bond angle around an un-typed atom to determine sp and sp^2 ^hybridized centers. 5-membered and 6-membered rings are checked for planarity to estimate aromaticity. Finally, atoms marked as unsaturated are checked for an unsaturated neighbor to give a double or triple bond. After this initial atom typing, known functional groups are matched, followed by aromatic rings, followed by remaining unsatisfied bonds based on a set of heuristics for short bonds, atomic electronegativity, and ring membership.

Atom typing is performed by "lazy evaluation," matching atoms against SMARTS patterns to determine hybridization, implicit valence, and external atom types. Atom type perception may be triggered by adding hydrogens (which requires determination of implicit and explicit valence), exporting to a file format that requires atom types, or as requested by the user. To minimize the amount of typing required, when importing from a format with atom types specified, a lookup table is used to translate between equivalent types.

An important part of atom typing is aromaticity detection and assignment of Kekulé bond orders (kekulization). In Open Babel, a central aromaticity model is used, largely matching the commonly used Daylight SMILES representation [1], but with added support for aromatic phosphorous and selenium. Potential aromatic atoms and bonds are flagged on the basis of membership in a ring system possibly containing 4n+2 π electrons. Aromaticity is established only if a well-defined valence bond Kekulé pattern can be determined. To do this, atoms are added to a ring system and checked against the 4n+2 π electron configuration, gradually increasing the size to establish the largest possible connected aromatic ring system. Once this ring system is determined, an exhaustive search is performed to assign single and double bonds to satisfy all valences in a Kekulé form. Since this process is exponential in complexity, the algorithm will terminate if more than 30 levels of recursion or 15 seconds are exceeded (which may occur in the case of large fused ring systems such as carbon nanotubes).

### Canonical Representation of Molecules

In general, for any particular molecular structure and file format, there are a large number of possible ways the structure could be stored; for example, there are N! ways of ordering the atoms in an MOL file. While each of the orderings encodes exactly the same information, it can be useful to define a canonical numbering of the atoms of a molecule and use this to derive a canonical representation of a molecule for a particular file format. For a zero-dimensional file format without coordinates, such as SMILES, the canonical representation could be used to index a database, remove duplicates or search for matches.

Open Babel implements a sophisticated canonicalization algorithm that can handle molecules or molecular fragments. The atom symmetry classes are the initial graph invariants and encode topological and chemical properties. A cooperative labeling procedure is used to investigate the automorphic permutations to find the canonical code. Although the algorithm is similar to the original Morgan canonical code [21], various improvements are implemented to improve performance. Most notably, the algorithm implements heuristics from the popular nauty package [22,23]. Another aspect handled by the canonical code is stereochemistry as different labelings can lead to different parities. This is further complicated by the possibility of symmetry-equivalent stereocenters and stereocenters whose configuration is interdependent. The full details will be the subject of a separate publication.

### Coordinate Generation in 2D and 3D

**Figure 1:**
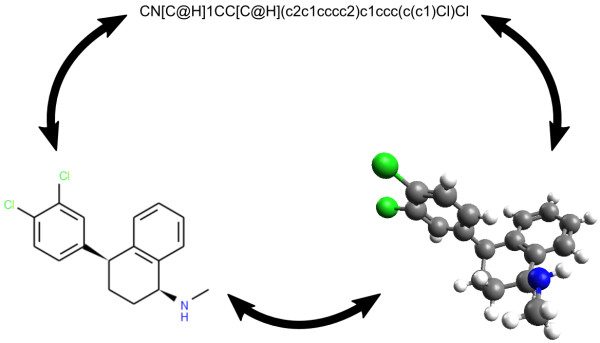
**Interconversion of 0D, 2D and 3D structures**. The structures shown are of sertraline, a selective serotonin reuptake inhibitor (SSRI) used in the treatment of depression. A SMILES string for sertraline is shown at the top; this can be considered a 0D structure (only connectivity and stereochemical information). From this, Open Babel can generate a 2D structure (bottom left, depicted by Open Babel) or a 3D structure (bottom right, depicted by Avogadro), and all of these can be interconverted.

Open Babel, version 2.3, has support for 2D coordinate generation (Figure 1) through the donation of code by Sergei Trepalin, based on the code used in the MCDL chemical structure editor [24-26]. The MCDL algorithm aims to layout the molecular structure in 2D such that all bond lengths are equal and all bond angles are close to 120°. The layout algorithm includes a small database of around 150 templates to help layout cages and large fragment cycles. To deal with the problem of overlapping fragments, the algorithm includes an exhaustive search procedure that rotates around acyclic bonds by 180°.

Coordinate generation in 3D was introduced in Open Babel version 2.2, and improved in version 2.3, to enable conversion from 0D formats such as SMILES to 3D formats such as SDF (Figure 1). The 3D structure generator builds linear components from scratch following geometrical rules based on the hybridization of the atoms. Single-conformer ring templates are used for ring systems. The template matching algorithm iterates through the templates from largest to smallest searching for matches. If a match is found, the algorithm continues but will not match any ring atoms previously templated except in the case of a single overlap (the two ring systems of a spiro group) or an overlap involving exactly two adjacent atoms (two fused ring systems). After an initial structure is generated, the stereochemistry (cis/trans and tetrahedral) is corrected to match the input structure. Finally, the energy of the structure is minimized using the MMFF94 forcefield [27-31] and a low energy conformer found using a weighted rotor search.

While the 3D structure builder produces reasonable conformations for molecules without rings or with ring systems for which a template exists, the results may be poor for molecules with more complex ring systems or organometallic species. Future work will be performed to compare the results of Open Babel with other programs with respect to both speed and the quality of the generated structures [32].

### Stereochemistry

A recent focus of Open Babel development has been to ensure robust translation of stereochemical information between file formats. This is particularly important when dealing with 0D formats as these explicitly encode the perceived stereochemistry. Open Babel 2.3 includes classes to handle cis/trans double bond stereochemistry, tetrahedral stereochemistry and square-planar stereochemistry (this last is still under development), as well as perception routines for 2D and 3D geometries, and routines to query and alter the stereochemistry.

The detection of stereogenic units starts with an analysis of the graph symmetry of the molecule to identify the symmetry class of each atom. However, given that a complete symmetry analysis also needs to take stereochemistry into account, this means that the overall stereochemistry can only be found iteratively. At each iteration, the current atom symmetry classes are used to identify stereogenic units. For example, a tetrahedral center is identified as chiral if it has four neighbors with different symmetry classes (or three, in the case where a lone pair gives rise to the tetrahedral shape).

### Forcefields

Molecular mechanics functions are provided for use with small molecules. Typical applications include energy evaluation or minimization, alone or as part of a larger workflow. The selection of implemented force fields allows most molecular structures to be used and parameters to be assigned automatically. The MMFF94(s) force field can be used for organic or drug-like molecules [27-31]. For molecules containing any element of the periodic table or complex geometry (i.e. not supported by MMFF94), the UFF force field can be used instead [33]. Recently, code implementing the GAFF force field [34,35] was also contributed and released as part of version 2.3. All of the forcefields allow the application of constraints on particular atom positions, or particular distances.

Several conformer searching methods have been implemented using the forcefields, all based on the "torsion-driving" approach. This approach involves setting torsion angles from a set of predefined allowed values for a particular rotatable bond. The most thorough search method implemented is a systematic search method, which iterates over all of the allowed torsion angles for each rotatable bond in the molecule and retains the conformer with the lowest energy. Since a systematic search may not be feasible for a molecule with multiple rotatable bonds, a number of stochastic search methods are also available: the random search method, which tries random settings for the torsion angles (from the predefined allowed values), and a weighted rotor search, a stochastic search method that converges on a low energy conformer by weighting particular torsion angles based on the relative energy of the generated conformer. With Open Babel 2.3, conformer search based on a genetic algorithm is also available which allows the application of filters (e.g. a diversity filter) and different scoring functions. This latter method can be used to generate a library of diverse conformers, or like the other methods to seek a low energy conformer [36].

## Implementation

### Technical Details

Open Babel is implemented in standards-compliant C++. This ensures support for a wide variety of C++ compilers (MSVC, GCC, Intel Compiler, MinGW, Clang), operating systems (Windows, Mac OS X, Linux, BSD, Windows/Cygwin) and platforms (32-bit, 64-bit). Since version 2.3, it is compiled using the CMake build system [37,38]. This is an open-source cross-platform build system with advanced features for dependency analysis. The build system has an associated unit test framework CTest, which allows nightly builds to be compiled and tested automatically with the results collated and displayed on a centralized dashboard [39].

To simplify installation Open Babel has as few external dependencies as possible. Where such dependencies exist, they are optional. For example, if the XML development libraries are not available, Open Babel will still compile successfully but none of the XML formats (such as Chemical Markup Language, CML) will be available. Similarly, if the Eigen matrix and linear algebra library is not found, any classes that require fast matrix manipulation (such as OBAlign, which performs least squares alignment) will not be compiled.

While the majority of the Open Babel library is written in C++, bindings have been developed for a range of other programming languages, including Java and the .NET platform, as well as the so-called "dynamic" scripting languages Perl, Python, and Ruby. These are automatically generated from the C++ header files using the SWIG tool. As described previously [40], in the case of Python an additional module is provided named Pybel that simplifies access to the C++ bindings. These interfaces facilitate development of web-enabled chemistry applications, as well as rapid development and prototyping.

### Code Architecture

**Figure 2:**
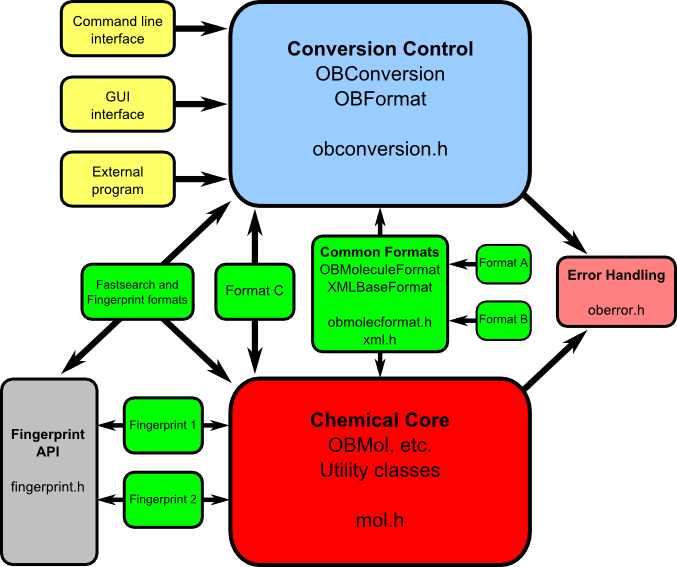
**Architecture of the Open Babel codebase**.

The Open Babel codebase has a modular design as shown in Figure 2. The goal of this design is threefold:

1. To separate the chemistry, the conversion process and the user interfaces reducing, as far as possible, the dependency of one upon another.

2. To put all of the code for each chemical format in one place (usually a single file) and make the addition of new formats simple.

3. To allow the format conversion of not just molecules, but also any other chemical objects, such as reactions.

The code base can be considered as consisting of the following modules (Figure 2):

• The Chemical Core, which contains OBMol etc. and has all of the chemical structure description and manipulation. This is the heart of the application and its API can be used as a chemical toolbox. It has no input/output capabilities.

• The Formats, which read and write to files of different types. These classes are derived from a common base class, OBFormat, which is in the Conversion Control module. They also make use of the chemical routines in the Chemical Core module. Each format file contains a global object of the format class. When the format is loaded the class constructor registers the presence of the class with OBConversion. This means that the formats are plugins - new formats can be added without changing any framework code.

• Common Formats include OBMoleculeFormat and XMLBaseFormat from which most other formats (like Format A and Format B in the diagram) are derived. Independent formats like Format C are also possible.

• The Conversion Control, which also keeps track of the available formats, the conversion options and the input and output streams. It can be compiled without reference to any other parts of the program. In particular, it knows nothing of the Chemical Core: mol.h is not included.

• The User Interface, which may be a command line application, a Graphical User Interface (GUI), or may be part of another program that uses Open Babel's input and output facilities. This depends only on the Conversion Control module (obconversion.h is included), but not on the Chemical Core or on any of the Formats.

• The Fingerprint API, as well as being usable in external programs, is employed by the fastsearch and fingerprint formats.

• The Fingerprints, which are bit arrays that describe an object and which facilitate fast searching. They are also built as plugins, registering themselves with their base class OBFingerprint which is in the Fingerprint API.

• Other features such as Forcefields, Partial Charge Models and Chemical Descriptors, although not shown in the diagram, are handled similarly to Fingerprints.

• The Error Handling can be used throughout the program to log and display errors and warnings.

### Extensible Interface

The utility of software libraries such as Open Babel depends on the ability of the design to be extended over time to support new functionality. To facilitate this, Open Babel implements a *plugin interface *for file formats, fingerprints, charge models, descriptors, "operators" and molecular mechanics force fields. This ensures a clean separation of the implementation of a particular plugin from the core Open Babel library code, and makes it easy for a new plugin (e.g. a new file format) to be contributed; all that is needed is a single C++ file and a trivial change to one of the build files. The operator plugins provide a very general mechanism for operating on a molecule (e.g. energy minimization or 3D coordinate generation) or on a list of molecules (e.g. filtering or sorting) after reading but before writing.

Plugins are dynamically loaded at runtime. This decreases the overall disk and memory footprint of Open Babel, allowing external developers to choose particular functionality needed for their application and ignore other, less relevant features. It also allows the possibility of a third-party distributing plugins separately to the Open Babel distribution to provide additional functionality.

### Open-Source License and Open Development

Open Babel is open-source software, which offers end users and third-party developers a range of additional rights not granted by proprietary chemistry software. Open-source software, at its most basic level, grants users the rights to study how their software works, to adapt it for any purpose or otherwise modify it, and to share the software and their modifications with others. In this sense, Open Source functions in similar ways to the processes of open peer review, publication, and citation in science. The rights granted by open source licenses largely coincide with the norms of scientific ethics to enable verifiability, repeatability, and building on previous results and theories.

**Figure 3:**
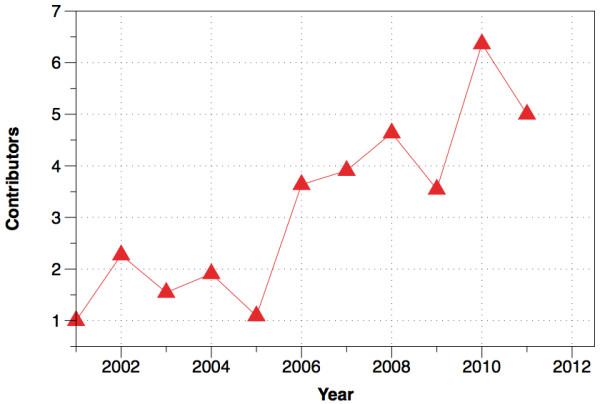
**Number of contributors over time**. Note that this graph only includes developers who directly commited code to the Open Babel source code repository, and does not include patches provided by users.

Beyond these rights, Open Babel (like most other open-source projects) offers open development -- that is, all development occurs in public forums and with public code repositories. This results in greater input from the community as any user can easily submit bug reports or feature suggestions, get involved in discussions on the future direction of Open Babel or even become a developer him/herself. In practice, the number of active contributors has increased over time through this level of open, public development (Figure 3). Moreover, it means that the development of the code is completely transparent and the quality of the software is available for public scrutiny. Indeed, since its inception, over 658 bugs have been submitted to the public tracker and fixed [41].

### Validation and Testing

Open Babel includes an extensive test suite comprising 60 different test programs each with tens to hundreds of tests. In early 2010, a nightly build infrastructure and dashboard was put in place with support from Kitware, Inc. This has greatly improved code quality by catching regressions, and also ensures that the code compiles cleanly on all platforms and compilers supported by Open Babel. Some examples of tests that are run each night are:

(1) The MMFF94 forcefield code is tested against the MMFF94 validation suite.

(2) The OBAlign class, which was developed using Test-Driven Development (TDD) methodology, is run against its test suite.

(3) Handling of symmetry is validated by converting several test cases between SMILES, 2D and 3D SDF, and InChI (there are also several test programs with unit tests for the individual stereo classes in the API).

(4) The SMARTS parser is tested using over 250 valid and invalid SMARTS patterns, and the SMARTS matcher is tested using 125 basic SMARTS patterns.

(5) The LSSR (Least Set of Smallest Rings) code is tested for invariance against changing the atom order for a series of polycyclic molecules.

**Figure 4:**
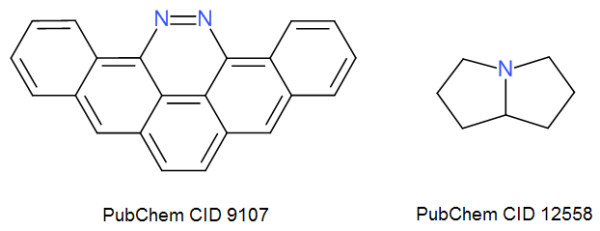
**The two failures found in the validation test for reading/writing SMILES**.

Recently the development team has placed a major focus on increasing the robustness of file format translation particularly in relation to the commonly used SMILES and MDL Molfile formats. Translating between these formats requires accurate stereochemistry perception, inference of implicit hydrogens, and kekulization of delocalized systems. While it is difficult to ensure that any complex piece of code is free of bugs, and Open Babel is no exception, validation procedures can be carried out to assess the current level of performance and to find additional test cases that expose bugs. The following procedure was used to guide the rewriting of stereochemistry code in Open Babel, a project that began in early 2009. Starting with a dataset of 18,084 3D structures from PubChem3D as an SDF file, we compared the result of (a) conversion to SMILES, followed by conversion of that to Canonical SMILES to (b) conversion directly to Canonical SMILES. This procedure can be used to flush out errors in reading the original SDF file, reading/writing SMILES (either due to stereochemistry errors or kekulization problems), and is also a test (to some extent) of the canonicalization code. At the time of starting this work (March 2009), the error rate found was 1424 (8%); by Oct 2009, combined work on stereochemistry, kekulization and canonicalization had reduced this to 190 (~1%), and continued improvements have reduced the number of errors down to two (shown in Figure 4) for Open Babel 2.3.1 (~0.01%). The first failure is due to a kekulization error in a polycyclic aromatic molecule incorporating heteroatoms: (a) gave c1ccc2c(c1)c1[nH][nH]c3c4c1c(c2)ccc4cc1c3cccc1 while (b) gave c1ccc2c(c1)c1nnc3c4c1c(c2)ccc4cc1c3cccc1. This error led to confusion over whether or not the aromatic nitrogens have hydrogens attached (they do not). The second failure involves confusion over the canonical stereochemistry at a bridgehead carbon: (a) gave C1CN2[C@@H](C1)CCC2 while (b) gave C1CN2[C@H](C1)CCC2. This is actually a meso compound and so both SMILES strings are correct and represent the same molecule. However the canonicalization algorithm should have chosen one stereochemistry or the other for the canonical representation.

**Figure 5:**
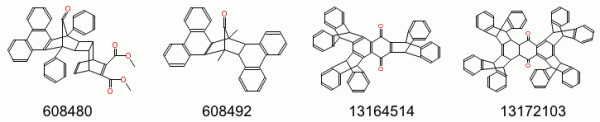
**The four failures found in the validation test for canonicalization**.

Another area of focus was the canonicalization algorithm, which can be used to generate canonical SMILES as well as other formats. The algorithm can be tested by ensuring that the same canonical SMILES string is obtained even when the order of atoms in a molecule is changed (while retaining the same connection table). The test stresses all areas of the library, including aromaticity perception, kekulization, stereochemistry, and canonicalization. The development of the canonicalization code in Open Babel was guided by applying this test to the 5,151,179 molecules in the eMolecules catalogue (dated 2011-01-02) with 10 random shuffles of the atom order. At the time of the Open Babel 2.2.3 release, there were 24,404 failures of the canonicalization algorithm; this has now been reduced to only four (shown in Figure 5, < 0.001%). The Open Babel nightly test suite ensures that this test passes for a number of problematic molecules. Although the canonicalization algorithm is still not perfect, we believe that the current level of performance (99.99992% success on the eMolecules catalogue) is acceptable for general use and with time we intend to improve performance further.

Given that the error rate for canonicalization and handling of stereochemistry is now quite low, the next area of focus for the Open Babel development team is to improve the handling of implicit valence for "unusual atoms." This is particularly important for organometallic species and inorganic complexes.

## Using Open Babel

### Applications

The Open Babel package is composed of a set of user applications as well as a programming library. The main command line application provided is *obabel *(a small upgrade on the earlier *babel*), which facilitates file format conversion, filtering (by SMARTS, title, descriptor value, or property field), 3D or 2D structure generation, conversion of hydrogens from implicit to explicit (and vice versa), and removal of small fragments or of duplicate structures. A number of features are provided to handle multi-molecule file formats (such as SDF or MOL2) and to use or manipulate the information in property fields and molecule titles. Here is an example of using *obabel *to convert from SDF format to SMILES:

obabel inputmols.sdf -O outputmols.smi

A more complicated use would be to extract all molecules in an SDF file whose titles start with "active":

obabel inputmols.sdf -aT -o copy -O outputmols.sdf --filter "title='active*'"

The *copy *format specified by "-o copy" is a utility format that copies the exact contents of the input file (for the filtered molecules) directly to the output, without perception or interpretation. The "-aT" indicates that only the title of the input SDF file should be read; full chemical perception is not required.

**Figure 6:**
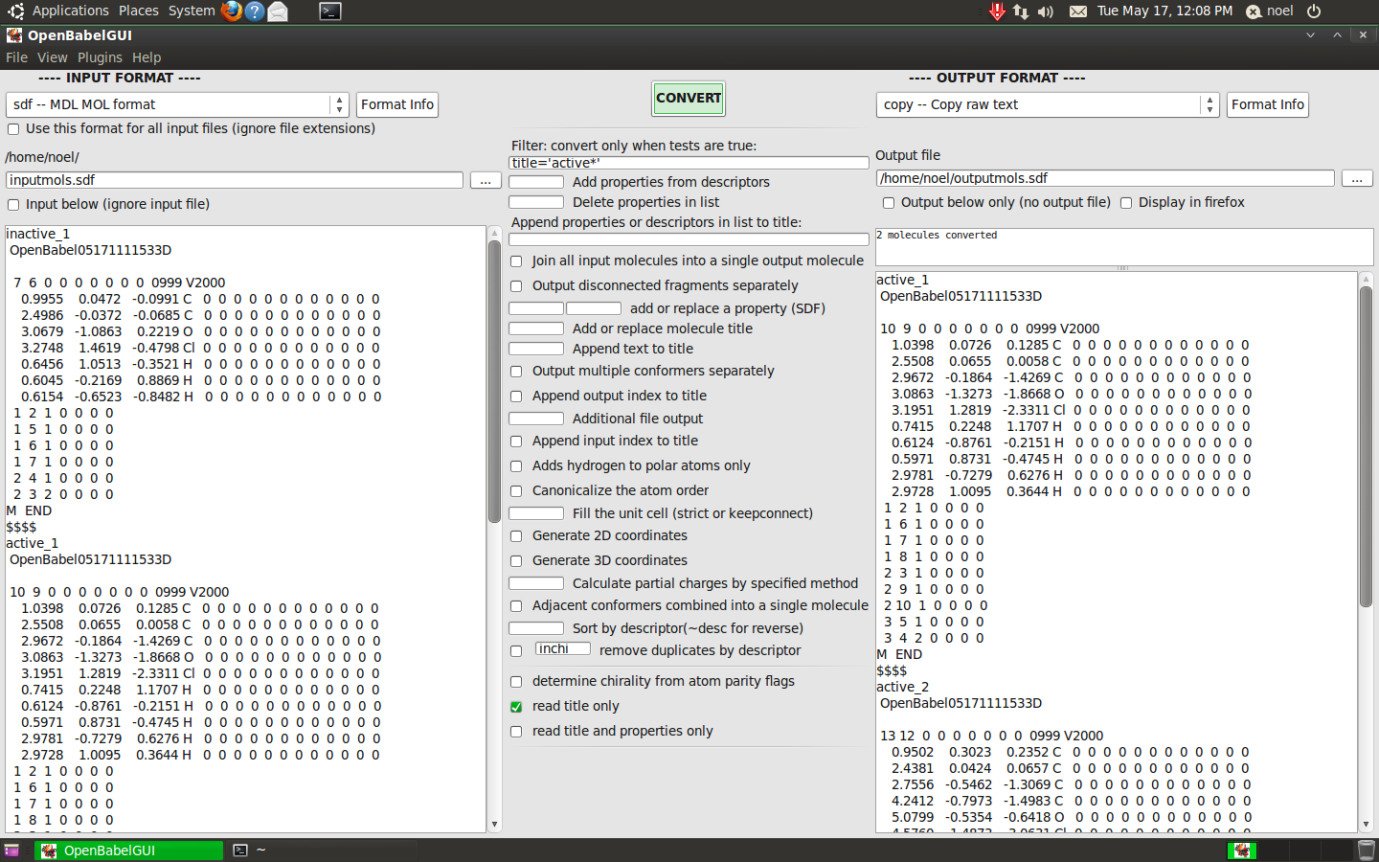
**Screenshot of the Open Babel GUI**. In the screenshot, the Open Babel GUI is running on Bio-Linux 6.0, an Ubuntu derivative.

The Open Babel graphical user interface (GUI) provides the same functionality. Figure 6 is a screenshot of the GUI carrying out the same filtering operation described in the *obabel *example above. The left panel deals with setting up the input file, the right panel handles the output and the central panel is for setting conversion options. Depending on whether a particular option requires a parameter, the available options are displayed either as check boxes or as text entry boxes. These interface elements are generated dynamically directly from the text description and help text provided by each format plugin.

### Programming Library

The Open Babel library allows users to write chemistry applications without worrying about the low-level details of handling chemical information, such as how to read or write a particular file format, or how to use SMARTS for substructure searching. Instead, the user can focus on the scientific problem at hand, or on creating a more easy-to-use interface (e.g. a GUI) to some of Open Babel's functionality. The Open Babel API (Application Programming Interface) is the set of classes, methods and variables provided by Open Babel to the user for use in programs. Documentation on the complete API (generated using Doxygen [42]) is available from the Open Babel website [43], or can be generated from the source code.

The functionality provided by the Open Babel library is relied upon by many users and by several other software projects, with the result that introducing changes to the API would cause existing software to break. For this reason, Open Babel strives to maintain API stability over long periods of time, so that existing software will continue to work despite the release of new Open Babel versions with additional features, file formats and bug fixes. Open Babel uses a version numbering system that indicates how the API has changed with every release:

• Bug fix releases (e.g. 2.0.0 versus 2.0.1) do not change API at all

• Minor version releases (e.g. 2.0 versus 2.1) will add to the API, but will otherwise be backwards-compatible

• Major version releases (e.g. 2 versus 3) are not backwards-compatible, and have changes to the API (including removal of deprecated classes and functions)

**Figure 7:**
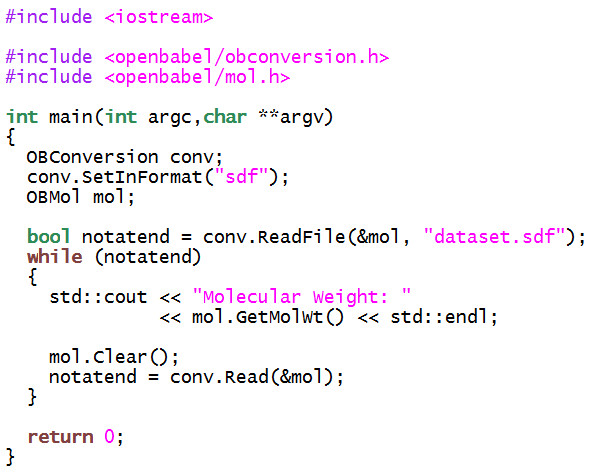
**Example C++ program that uses the Open Babel library**. The program prints out the molecular weight of each molecule in the SDF file "dataset.sdf".

**Figure 8:**
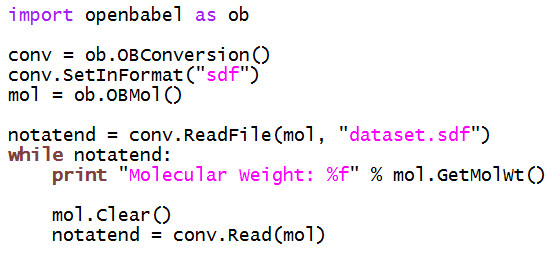
**Example Python program that uses the Open Babel library**. The program prints out the molecular weight of each molecule in the SDF file "dataset.sdf".

Figure 7 shows an example C++ program that uses the two main classes OBConversion and OBMol to print out the molecular weight of all of the molecules in an SDF file. This could be used, for example, to investigate differences in the molecular weight distribution between two databases. The same program is shown in Figure 8 but implemented using the Python bindings.

### Examples of Use

Open Babel has already been referenced over 400 times for various uses. The most common use of Open Babel is through the *obabel *command line application (or the corresponding graphical user interface) for the interconversion of chemical file formats. Such conversions may also involve the calculation or inference of additional molecular information or application of a filter. Some published examples of these include the following:

• interconversion of chemical file formats or representations [44-47]

• addition of hydrogens [48-50]

• generation of 3D molecular structures [51-53]

• calculation of partial charges [54,55]

• generation of molecular fingerprints [56-59]

• removal of duplicate molecules from a dataset [60]

• calculation of MOL2 atom types [61]

An interesting example that shows how a particular chemical representation may be used to facilitate a scientific study is the crystallographic study of Fábián and Brock who used Open Babel to generate InChI strings for molecules in the Cambridge Structural Database [62]. Exploiting the fact that InChIs of enantiomers are identical expect at the enantiomer sublayer ("/m0" or "/m1"), they used the InChIs as part of a workflow to identify kryptoracemates (a class of racemic crystals where the enantiomers are not related by space-group symmetry) in the database.

To implement new methods, or access additional molecular information, it is necessary to use the Open Babel library directly either from C++ or using one of the supported language bindings. Some examples of published studies that have done this include the following:

• Dehmer *et al. *implemented molecular complexity measures based on information theory [63].

• Langham and Jain developed a model for chemical mutagenicity based on atom pair features [64].

• Fontaine *et al. *implemented a method, anchor-GRIND, that uses an anchor point of a molecular scaffold to compare molecular interaction fields when different substituents are present [65].

• Konyk *et al. *have developed a plugin for Open Babel that adds support for the Web Ontology Language (OWL) to allow automated reasoning about chemical structures [66].

• Kogej *et al. *(AstraZeneca) implemented a 3-point pharmacophore fingerprint called TRUST [67].

• Many other examples exist [68-71].

The vital role that a cheminformatics toolkit plays in the development of scientific resources is shown by Tables 1 and 2. Table 1 lists examples of stand-alone applications or programming libraries that rely on Open Babel, either calling the library directly or via one of the command-line executables. Table 2 contains examples of web applications and databases that either use Open Babel on the server or where Open Babel was used in the preparation of the data.

**Table 1:** Software applications and libraries that use Open Babel

Name	Description	Reference	Web page
**Avogadro**	GUI for molecular modelling and computational chemistry	G. Hutchison M. Hanwell	http://avogadro.openmolecules.net/
**cclib**	Parse computational chemistry output files	[72]	http://cclib.sf.net/
**CCP1GUI**	GUI for computational chemistry	Jens Thomas	http://www.cse.scitech.ac.uk/ccg/software/ccp1gui
**ChemAzTech**	Manage a chemical laboratory database	Rémy Dernat	http://chemaztech.sf.net/
**ChemSpotlight**	Chemistry file indexer for MacOSX	G. Hutchison	http://chemspotlight.openmolecules.net/
**ChemT**	GUI for generating combinatorial libraries	Rui Abreu	http://www.esa.ipb.pt/~ruiabreu/chemt
**ChemTool**	2D molecular drawing	[73]	http://ruby.chemie.uni-freiburg.de/~martin/chemtool
**CMDF**	Library for handling and preparing multi-scale multi-paradigm simulations	[74]	http://web.mit.edu/mbuehler/www/research/CMDF/CMDF.htm
**Confab**	Systematically generate conformers	[36]	http://confab.googlecode.com/
**DockoMatic**	Automate the preparation and analysis of AutoDock runs	[75]	http://sf.net/projects/dockomatic/
**DOVIS 2.0**	Automate the preparation and analysis of AutoDock runs	[76]	http://www.bhsai.org/dovis.html
**FAF-Drugs2**	ADMET filtering of molecular datasets	[77]	http://www.mti.univ-paris-diderot.fr/fr/downloads.html
**FMiner2**	Large-scale chemical graph mining based on backbone refinement classes	[78,79]	http://www.maunz.de/wordpress/bbrc
**Ghemical**	GUI for computational chemistry	Tommi Hassinen	http://www.uku.fi/~thassine/projects/ghemical
**Gnome Chemistry Utils**	2D chemical editor, 3D viewer, chemical calculator and periodic table for Linux	Jean Bréfort	http://gchemutils.nongnu.org/
**iBabel**	MacOSX interface to Open Babel and other Open chemistry tools	Chris Swain	http://homepage.mac.com/swain/Sites/Macinchem/page65/ibabel3.html
**Kalzium**	GUI showing information on the periodic table of the elements	Carsten Niehaus	http://edu.kde.org/kalzium/
**Lazar**	Lazy Structure-Activity Relationships for toxicity prediction	[80]	http://www.in-silico.de/software/
**Molekel**	GUI for computational chemistry	Ugo Varetto	http://molekel.cscs.ch/
**molsKetch**	2D chemical editor	Harm van Eersel	http://molsketch.sf.net/
**MyChem**	Chemistry extension to the MySQL database	J. Pansanel	http://mychem.sf.net/
**NanoEngineer-1**	Computer-aided design for the nanoscale	Nanorex, Inc.	http://nanoengineer-1.net/
**NanoHive-1**	Simulator for the study, experimentation, and development of nanotech entities	Brian Helfrich	http://www.nanohive-1.org/
**OpenMD**	Open Source molecular dynamics engine	[81]	http://openmd.net/
**Open3DQSAR**	High-throughput chemometric analysis of molecular interaction fields	[82,83]	http://www.open3dqsar.org/
**OSRA**	Extracts chemical structures from images	[84]	http://osra.sf.net/
**PgChem**	Chemistry extension to the PostgreSQL database	Ernst-Georg Schmidt	http://pgfoundry.org/projects/pgchem
**Pharao**	Pharmacophore discovery and searching	Silicos NV	http://www.silicos.be/
**Pharmer**	Pharmacophore searching	[85]	http://smoothdock.ccbb.pitt.edu/pharmer
**Piramid**	Shape-based alignment of molecules	Silicos NV	http://www.silicos.be/
**PyADF**	Library for handling and preparing quantum mechanical multi-scale simulations	[86]	http://www.ipc.kit.edu/cfn-ysg/158.php
**PyRx**	GUI for virtual screening with protein-ligand docking	Sargis Dallakyan	http://pyrx.scripps.edu/
**QMForge**	GUI for analysing results of quantum chemistry calculations	[72]	http://qmforge.sf.net/
**RMG**	Reaction Mechanism Generator	[87]	http://rmg.sf.net/
**Sci3D**	Interactive visualization of 3D models of scientific data, such as molecular structures and surfaces	T.J. O'Donnell	http://sci3d.sf.net/
**Sieve**	Filter molecules from datasets	Silicos NV	http://www.silicos.be/
**SMIREP**	Generation of fragment-based structure-activity relationships	[88]	http://www.karwath.org/systems/smirep.html
**Stripper**	Extract molecular scaffolds	Silicos NV	http://www.silicos.be/
**Toxtree**	Toxic hazard estimation using decision trees	Ideaconsult Ltd.	http://toxtree.sf.net/
**V_Sim**	Visualize atomic structures such as crystals and grain boundaries	Damien Caliste	http://inac.cea.fr/L_Sim/V_Sim/index.en.html
**WebBabel**	Web application for file format conversion	T.J. O'Donnell	http://webbabel.sf.net/
**XDrawChem**	2D molecular editor	Bryan Herger	http://xdrawchem.sf.net/
**XtalOpt**	Extension to Avogadro for crystal-structure prediction	[89]	http://xtalopt.openmolecules.net/
**YASARA**	GUI for molecular graphics, modeling and simulation	Elmar Krieger	http://www.yasara.org/
**ZODIAC**	GUI for molecular modelling and docking	[90]	http://www.zeden.org/

**Table 2:** Web applications and databases that use Open Babel

Name	Description	Reference	Web page
**ChemDB**	Database of small molecules	[91]	http://cdb.ics.uci.edu/
**Cheméo**	Chemical structure and property search engine	Céondo Ltd	http://www.chemeo.com/
**ChemMine Tools**	Web application for analysing and clustering small molecules	[92]	http://chemmine.ucr.edu/
**eMolecules**	Chemical vendor search engine	eMolecules.com	http://emolecules.com/
**FragmentStore**	Database for comparison of fragments found in metabolites, drugs and toxic compounds	[93]	http://bioinf-applied.charite.de/fragment_store/
**Frog2**	FRee Online druG 3D conformation generation	[94]	http://bioserv.rpbs.univ-paris-diderot.fr/cgi-bin/Frog2
**hBar Lab**	Web application providing on-demand access to computer-aided chemistry	hBar Solutions ApS	https://www.hbar-lab.com/
**IUPHAR-DB**	Database of human drug targets and their ligands	[95]	http://www.iuphar-db.org/
**OpenCDLig**	Web application for sharing resources about cyclodextrin/ligand complexes	[96]	https://kdd.di.unito.it/casmedchem/
**PSMDB**	Protein - Small-Molecule Database	[97]	http://compbio.cs.toronto.edu/psmdb/
**SambVca**	Web application for calculation of buried volume of organometallic ligands	[98]	https://www.molnac.unisa.it/OMtools/sambvca.php
**ScafBank**	Database of molecular scaffolds	[99]	http://202.127.30.184:8080/scafbank.html
**SMARTCyp**	Web application for prediction of sites of cytochrome P450 mediated metabolism	[100]	http://www.farma.ku.dk/smartcyp/
**sMol Explorer**	Web application for exploring small-molecule datasets	[101]	http://www3a.biotec.or.th/isl/index.php/smol-explorer
**SuperImposé**	Web application for structural similarity between ligands, binding sites or proteins	[102]	http://farnsworth.charite.de/superimpose-web/
**SuperToxic**	Database of toxic compounds	[103]	http://bioinformatics.charite.de/supertoxic/
**SuperSite**	Detailed information on, and comparisons of, protein-ligand binding sites	[104]	http://bioinf-tomcat.charite.de/supersite/
**SuperSweet**	Database of natural and artificial sweeteners	[105]	http://bioinf-applied.charite.de/sweet/
**STITCH2**	Chemical-protein interactions	[106]	http://stitch.embl.de/
**VCCLAB**	Virtual Computational Chemistry Laboratory	[107]	http://www.vcclab.org/
**wwLigCSRre**	Web application that performs ligand-based screening using 3D similarity	[108]	http://bioserv.rpbs.univ-paris-diderot.fr/Help/wwLigCSRre.html

## Conclusions

In November 2011, Open Babel will mark 10 years of existence as an independent project, and for the first time, we have discussed its development and features. As shown by more than 400 citations, it has become an essential tool for handling the myriad of molecular file formats encountered in diverse branches of chemistry. While more work remains to be done, through validation processes such as those described above and the recent introduction of a nightly build and testing framework, we aim to improve the quality and robustness of the toolkit with each new release.

Looking forward to the future, one of the goals of the project is to extend support to molecules that currently are not handled very well by existing cheminformatics toolkits. Typically toolkits focus on the types of molecules of principal importance to the pharmaceutical industry, namely stable organic molecules comprising wholly of 2-center 2-electron covalent bonds. Molecules outside this set - such as radicals, organometallic and inorganic molecules, molecules with coordinate bonds or 3-center 2-electron bonds - are poorly supported in general. Future releases of Open Babel will provide substantially improved handling of such species. We also seek to improve speed and coverage of important methods such as structure generation, kekulization and canonicalization.

Open Babel is freely available from http://openbabel.org, and new community members are very welcome (users, developers, bug reporters, feature requesters). For information on how to use Open Babel, please see the documentation at http://openbabel.org/docs and the API documentation at http://openbabel.org/api.

## Availability and Requirements

**Project Name: **Open Babel

**Project home page: **http://openbabel.org

**Operating system(s): **Cross-platform

**Programming language: **C++, bindings to Python, Perl, Ruby, Java, C#

**Other requirements (if compiling): **CMake 2.4+

**License: **GNU GPL v2

**Any restrictions to use by non-academics: **None

## Competing interests

The authors declare that they have no competing interests.

## Authors' contributions

GRH is the lead developer of the Open Babel project. CAJ, CM, MB, NMOB, and TV are developers of Open Babel. All authors read and approved the final manuscript.
